# Supplementary material for: Radio emissions reveal Alfvénic activity and electron acceleration prior to substorm onset
Source: Nat Commun. 2025 Nov 26;16:10553. doi: 10.1038/s41467-025-65580-8 (PMC12657864; doi:10.1038/s41467-025-65580-8)
Supplement: Supplementary file 1 — Supplementary Information [file 41467_2025_65580_MOESM1_ESM.pdf]

Supplementary Information for

Radio emissions reveal Alfvénic activity and electron acceleration prior to substorm onset

S.Y. Wu[1*](吴思远), D. K. Whiter[1], L. Lamy[2, 3], M. M. Wang[4](王蒙蒙), P. Zarka[3], C. M. Jackman[5], S. Y. Ye[6] (叶生毅), J. E. Waters[3], A. R. Fogg[5], S. B. Mende[7], N. Kaweeyanun[1], Y. Kasaba[8] (笠羽 康正), S. Kurita[9], H. Kojima[9], L. J. Paxton[10], Y. Kasahara[11], Y. Miyoshi[12, 13], A. Shinbori[12] (新堀 淳樹), F. Tsuchiya[8]

[1]School of Physics and Astronomy, University of Southampton, UK

[2]Aix Marseille University, CNRS, CNES, LAM, Marseille, France

[3]LIRA, Observatoire de Paris, CNRS, PSL, Sorbonne Université, Université Paris Cité, Meudon, France

[4]Swedish Institute of Space Physics, Uppsala, Sweden

[5]School of Cosmic Physics, DIAS Dunsink Observatory, Dublin Institute for Advanced Studies, Dublin, Ireland

[6]Department of Earth and Space Sciences, Southern University of Science and Technology, Shenzhen, People's Republic of China

[7]Space Science Laboratory, University of California, Berkeley, USA

[8]Planetary Plasma and Atmospheric Research Center, Graduate School of Science, Tohoku University, Aoba, Sendai, Japan

[9]Research Institute for Sustainable Humanosphere, Kyoto University, Uji, Japan

[10]The Johns Hopkins University Applied Physics Laboratory, Laurel, USA

[11]Graduate School of Natural Science and Technology, Kanazawa University, Kanazawa, Japan

[12]Institute for Space-Earth Environmental Research, Nagoya University Graduate School of Engineering, Nagoya University, Nagoya, Japan

[13]Kyung Hee University, Swon, Korea

Correspondence and requests for materials should be addressed to: Siyuan Wu (Siyuan.Wu@soton.ac.uk)

Supplementary Information includes:

Supplementary Figs. 1-5

Supplementary Data 1-2 (See Source Data files)

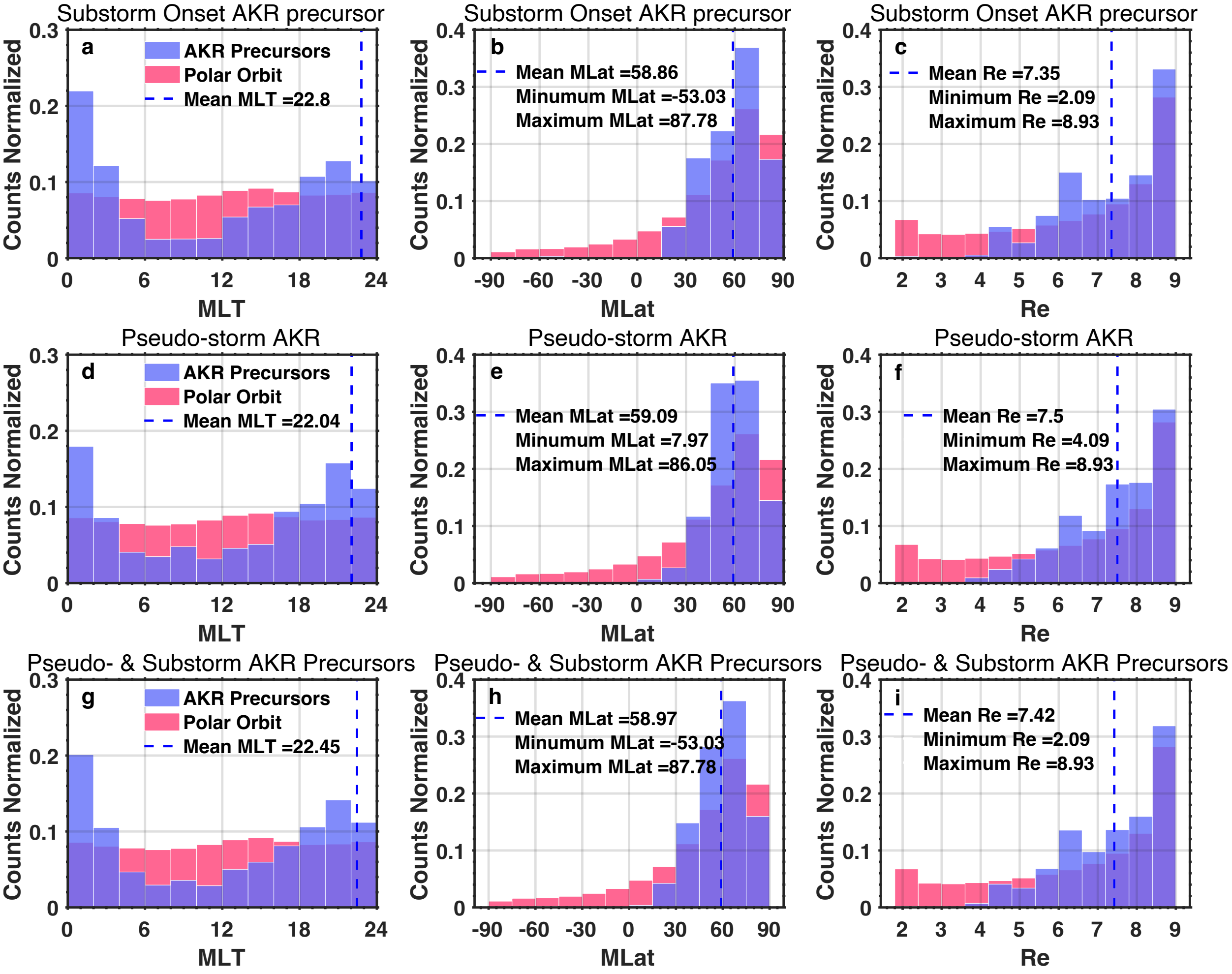

Supplementary Fig. 1. Distributions of AKR precursor observations in MLT, latitude, and radial distance. a-c, Normalized distributions of substorm-associated AKR precursors (blue) as functions of (a) magnetic local time (MLT), (b) magnetic latitude (MLat), and (c) radial distance (Re), compared to the corresponding coverage of the Polar spacecraft during 25 March 1996 to 16 September 1997 (red). d-f, Same as a-c, but for pseudo-storm–associated AKR precursors. g-i, Combined distributions of all AKR precursors (substorm- and pseudo-storm–associated) as functions of MLT, MLat, and Re. Source data are provided as a Source Data file.

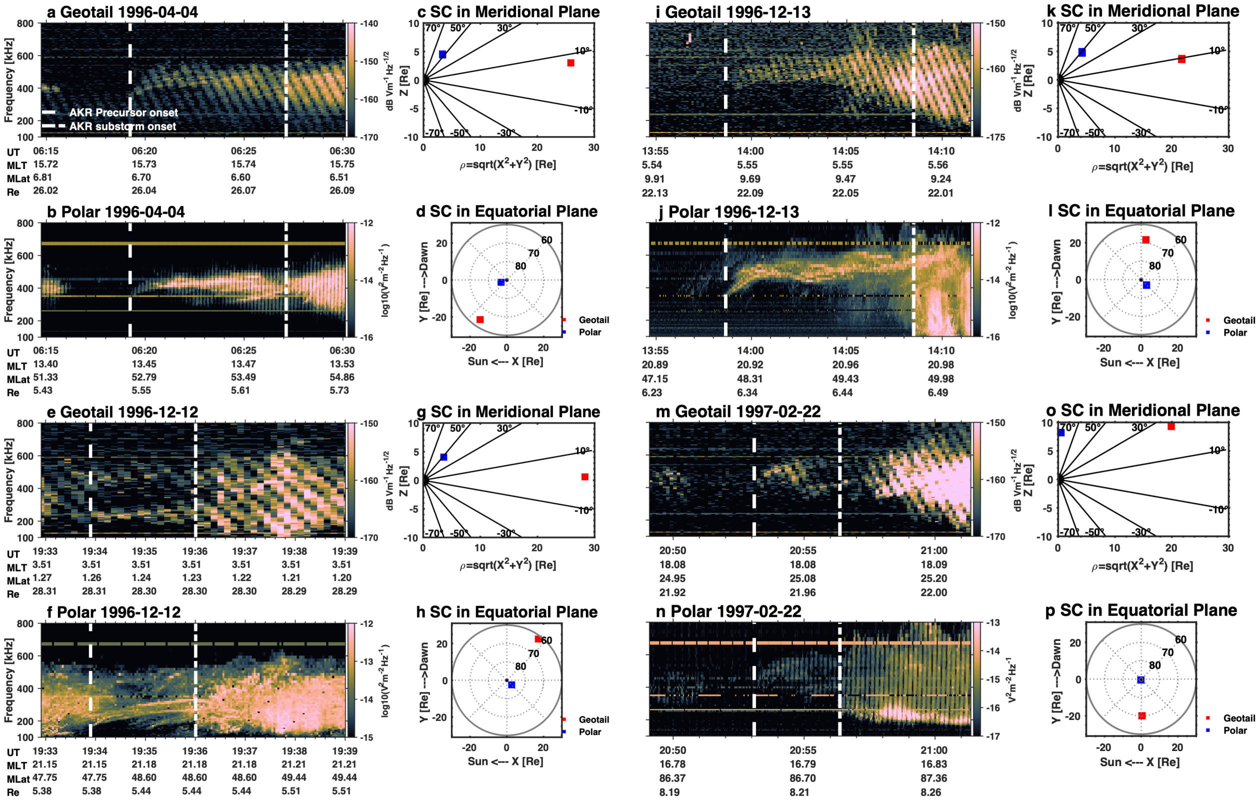

Supplementary Fig. 2. Simultaneous observations of AKR precursors by the Polar and Geotail spacecraft. a. AKR precursors observed by the Geotail PWI instrument, shown as a radio wave electric field spectrogram. The emissions of interest are highlighted between the two vertical white lines. The slanted (diagonal) lines seen in the spectrogram result from the receiver's frequency-sweeping logic and modulation introduced by the satellite's spin; these artifacts should be disregarded. b. Concurrent observations from the Polar PWI instrument, displayed in the same format as Panel a. The vertical modulations in the spectrogram are caused by the spacecraft spin. When data are recorded from antennas within the spin plane, these spin-related patterns are more prominent. In contrast, data recorded from antennas oriented perpendicular to the spin plane (as in Panels e and j) produce cleaner spectrograms with fewer spin-induced artifacts. c, Spacecraft positions projected onto the magnetic meridian plane. The Polar and Geotail locations are marked by blue and red squares, respectively. e-h, i-l, and m-p,

Three additional cases of simultaneous AKR precursor observations, each formatted similarly to a-d. To highlight the AKR precursor features, the color scales of the difference spectra are saturated at different levels. Source data are provided as a Source Data file.

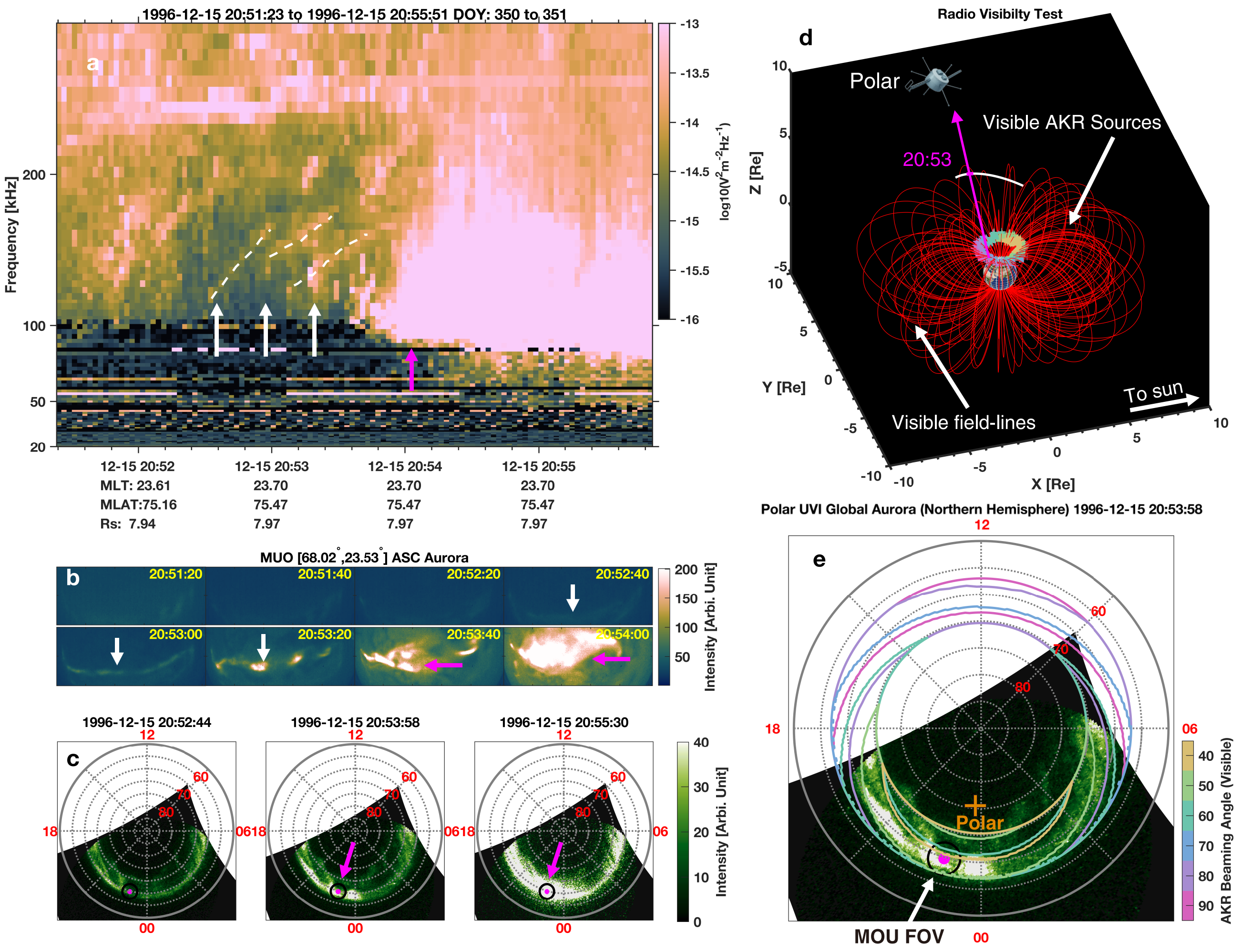

Supplementary Fig. 3. Simultaneous observations of AKR precursors and wave-like auroral forms: Case 3. a, Wave electric field spectrogram measured by the Polar PWI instrument from 20:51 to 20:55 UT on 15 December 1996. AKR precursors are indicated by white arrows; the substorm onset is marked by the pink arrow. b, Ground-based auroral image captured at the Muonio station (geographic latitude 68.02° N, longitude 23.53° E) using a 557.7 nm filter. c, Global auroral image taken by the Polar Ultraviolet Imager (UVI). The pink dot and black circle

mark the location and field of view of the Muonio all-sky camera. d, Radio visibility map during the time interval shown in a. The white curve indicates the Polar spacecraft trajectory; the pink segment (barely visible due to short duration) corresponds to the timing in a. Visible AKR source locations are color-coded by radio beaming angle, as indicated in the bottom-right color bar. Red lines trace magnetic field lines connecting visible AKR sources. e, Footprints of visible AKR sources projected onto a magnetic polar coordinate system. The background shows the global aurora in a green–white colormap from the Polar UVI. Colored contours outline the boundaries of visible AKR source footprints for different beaming angles. The pink dot and black circle indicate the Muonio station and camera field of view. The orange cross marks the magnetic footprint of Polar at 20:53:58 UT. Source data are provided as a Source Data file.

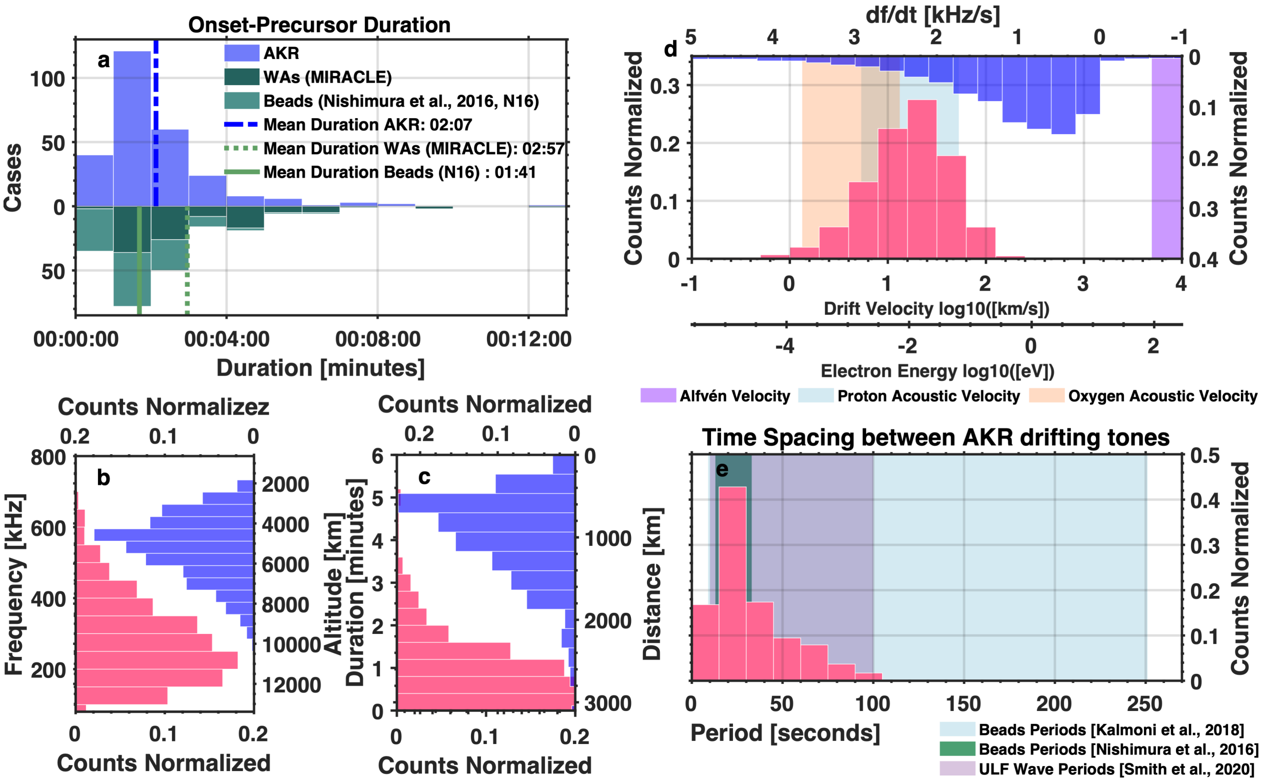

Supplementary Fig. 4. Statistical properties of substorm-associated AKR precursors and wave-like auroral forms. a, Distributions of event durations for AKR precursors (blue) and wave-

like aurora (green) associated with regular substorms. Dark green represents auroral events identified from MIRACLE observations in this study; light green shows the distribution of auroral bead durations from a previously published study (abbreviated as N16) [1]. b-e, Additional statistical properties of frequency-drifting tones identified in substorm-associated AKR precursors, shown in the same format as Fig. 5 of the main text. The color-shaded regions indicate the periods intervals highlighted in previously published results[1, 2, 3]. Source data are provided as a Source Data file.

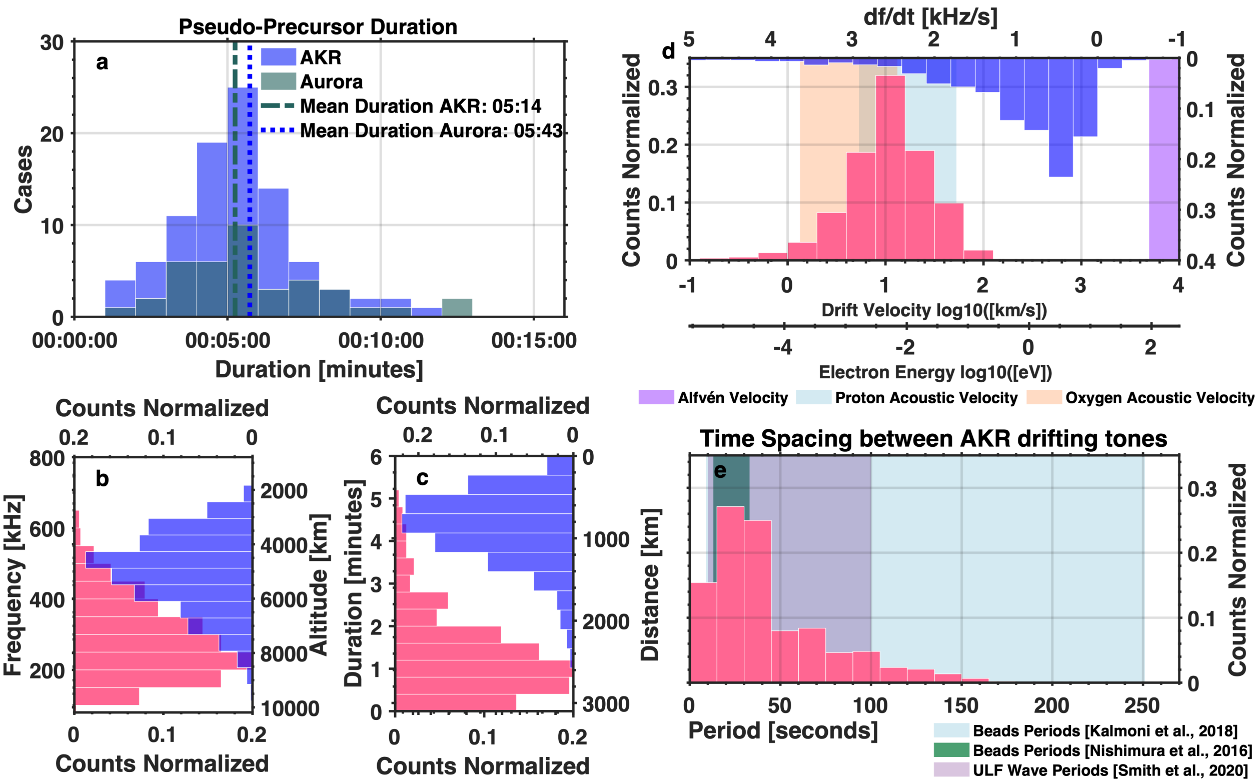

Supplementary Fig. 5. Statistical properties of pseudo-storm-associated AKR precursors and wave-like auroral forms. a-e, Statistical properties of frequency-drifting tones identified in pseudo-storm-associated AKR precursors, shown in the same format as Fig. 5 of the main text. Source data are provided as a Source Data file.

References

1. Nishimura, Y. et al. Statistical properties of substorm auroral onset beads/rays. J. Geophys. Res. Sp. Phys. 121, 8661–8676 (2016).
2. Kalmoni, N. M. E. et al. A diagnosis of the plasma waves responsible for the explosive energy release of substorm onset. Nat. Commun. 9, 4806 (2018).
3. Smith, A. W., Rae, I. J., Forsyth, C., Watt, C. E. J. & Murphy, K. R. On the Magnetospheric ULF Wave Counterpart of Substorm Onset. J. Geophys. Res. Sp. Phys. 125, e2019JA027573 (2020).
