# Supplementary material for: Radio emissions reveal Alfvénic activity and electron acceleration prior to substorm onset
Source: Nat Commun. 2025 Nov 26;16:10553. doi: 10.1038/s41467-025-65580-8 (PMC12657864; doi:10.1038/s41467-025-65580-8)
Supplement: Supplementary file 2 — Description of Additional Supplementary Files [file 41467_2025_65580_MOESM2_ESM.pdf]

Description of Additional Supplementary Files

Radio emissions reveal Alfvénic activity and electron acceleration prior to substorm onset

File Name: Supplementary Data 1

Description: List of AKR precursor events identified from the Polar PWI instrument. The columns labeled “No.”, “Start time”, “End time”, “Low Freq [Hz]”, and “Up Freq [Hz]” , “If drift emissions traced”, “If at least 1 frequency drifting tone”, “If drift emissions used for period calculations” correspond to the event number, the start time of each AKR precursor in the format “year-month-day Hour:Minute:Second”, the end time (AKR breakup time for substrom related event, and end time for the pseudo-storm related event), the lowest frequency during the event (in Hertz), the highest frequency during the event, if frequency drifting tone are traced in and used for df/dt analysis, if at least 1 frequency drifting tone is identified in this event (if no, it suggests strong background or noisy data), and if the identified drift emissions are used for period calculations, respectively.

File Name: Supplementary Data 2

Description: List of WAs events identified from the MIRACLE Cameras. The columns labeled “No.”, “Start time”, “End time”, “SOD”, “MUO”, “ABK”, “KIL”, and “KEV” indicate the event number, the start time of each auroral bead event in the format “year-month-day Hour:Minute:Second”, the end time (aurora breakup or poleward expansion time for substorm and aurora “dimming” or “disappearance” for pseudo-storm), and the stations where the WAs were observed. A value of 1 indicates the event was observed at that station, while 0 indicates it was not.
